# The role of the secretin/secretin receptor axis in inflammatory cholangiocyte communication via extracellular vesicles

**DOI:** 10.1038/s41598-017-10694-3

**Published:** 2017-09-11

**Authors:** Keisaku Sato, Fanyin Meng, Julie Venter, Thao Giang, Shannon Glaser, Gianfranco Alpini

**Affiliations:** 1 Research, Central Texas Veterans Health Care System, Temple, TX 76504 USA; 2 Department of Medicine, Texas A&M College of Medicine, Temple, TX 76504 USA; 3 Baylor Scott & White Digestive Disease Research Center, Baylor Scott & White Healthcare, Temple, TX 76504 USA; 4 Academic Research Integration, Baylor Scott & White Healthcare, Temple, TX 76504 USA

## Abstract

Small and large intrahepatic bile ducts consist of small and large cholangiocytes, respectively, and these cholangiocytes have different morphology and functions. The gastrointestinal peptide hormone, secretin (SCT) that binds to secretin receptor (SR), is a key mediator in cholangiocyte pathophysiology. Extracellular vesicles (EVs) are membrane-bound vesicles and cell-cell EV communication is recognized as an important factor in liver pathology, although EV communication between cholangiocytes is not identified to date. Cholangiocytes secrete proinflammatory cytokines during bacterial infection leading to biliary inflammation and hyperplasia. We demonstrate that cholangiocytes stimulated with lipopolysaccharide (LPS), which is a membrane component of gram-negative bacteria, secrete more EVs than cholangiocytes incubated with vehicle. These LPS-derived EVs induce inflammatory responses in other cholangiocytes including elevated cytokine production and cell proliferation. Large but not small cholangiocytes show inflammatory responses against large but not small cholangiocyte-derived EVs. Large cholangiocytes with knocked down either SCT or SR by short hairpin RNAs show reduced EV secretion during LPS stimulation, and EVs isolated from SCT or SR knocked down cholangiocytes fail to induce inflammatory reactions in control large cholangiocytes. This study identifies cholangiocyte EV communication during LPS stimulation, and demonstrates that the SCT/SR axis may be important for this event.

## Introduction

Intrahepatic cholangiocytes are heterogeneous epithelial cells that line a network of bile ducts, the biliary epithelium^1^. Cholangiocytes exposed to mediators of inflammation such as bacterial endotoxin or lipopolysaccharide (LPS) proliferate and secrete proinflammatory cytokines leading to biliary hyperplasia and inflammation, which are common characteristics of human cholangiopathies^2–4^. LPS-induced proliferating cholangiocytes secrete interleukin-6 (IL-6) and other proinflammatory cytokines leading to biliary damage and inflammation suggesting that cholangiocytes are primary target cells for chronic cholestatic liver diseases^5, 6^.

The gastrointestinal peptide hormone, secretin (SCT), is secreted by S cells of the duodenum as well as cholangiocytes^7^. SCT performs as a paracrine factor which binds to secretin receptor (SR) located on the basolateral domain of cholangiocytes leading to enhanced cholangiocyte proliferation and ductal secretion in a cyclic adenosine 3′,5′-monophosphate (cAMP)-dependent fashion during biliary damage^7–10^. SCT stimulates cholangiocyte proliferation by downregulating the expression of the microRNAs, 125b and let7a, and knockout of SR inhibits cholangiocyte proliferation and liver fibrosis during biliary damage indicating that the SCT/SR axis plays an important role in cholestatic liver injury^7, 11, 12^.

Cholangiocytes are morphologically and functionally heterogeneous with different diameter and protein expression between small and large cholangiocytes^9, 13–17^. Large, but not small cholangiocytes express SR and secrete water and HCO_3_^−^ following SCT stimulation, and respond to experimental cholestatic liver injury, such as bile duct ligation^8, 15^. Small cholangiocytes can differentiate into large cholangiocytes in a Ca^2+^-dependent pathway and it is suggested that small cholangiocytes are hepatic progenitor cells^9, 10, 18, 19^. However, detailed roles and associations between small and large cholangiocytes during cholestatic liver injury still need to be elucidated.

Extracellular vesicles (EVs) are membrane-bound vesicles released by various types of cells and are recognized to play a key role in liver pathology^20, 21^. Cholangiocarcinoma cell-derived EVs increase cytokine release including IL-6 from mesenchymal stem cells as well as phenotypic changes with fibrogenesis activity suggesting that the cell-cell communication via EVs may be a key for the progression of liver diseases^22^. Cholangiocytes interact with biliary EVs using primary cilia, and biliary EVs inhibit cholangiocyte proliferation suggesting that cholangiocyte homeostasis is controlled by biliary EVs^23^. However, the cell-cell communication between cholangiocytes via EVs and its function in cholestatic liver injury is largely unknown. We hypothesize about cell-cell communication between cholangiocytes during the events that LPS induces cholangiocyte proliferation and cytokine expression as described before^5, 6^. This study aimed to identify cell-cell communication between specific cholangiocyte subpopulations and also determine functional roles of the SCT/SR axis as well as cholangiocyte heterogeneity during LPS-induced inflammatory EV communication.

## Results

### LPS stimulation increased EV secretion from human H69 cholangiocytes

**Figure 1:**
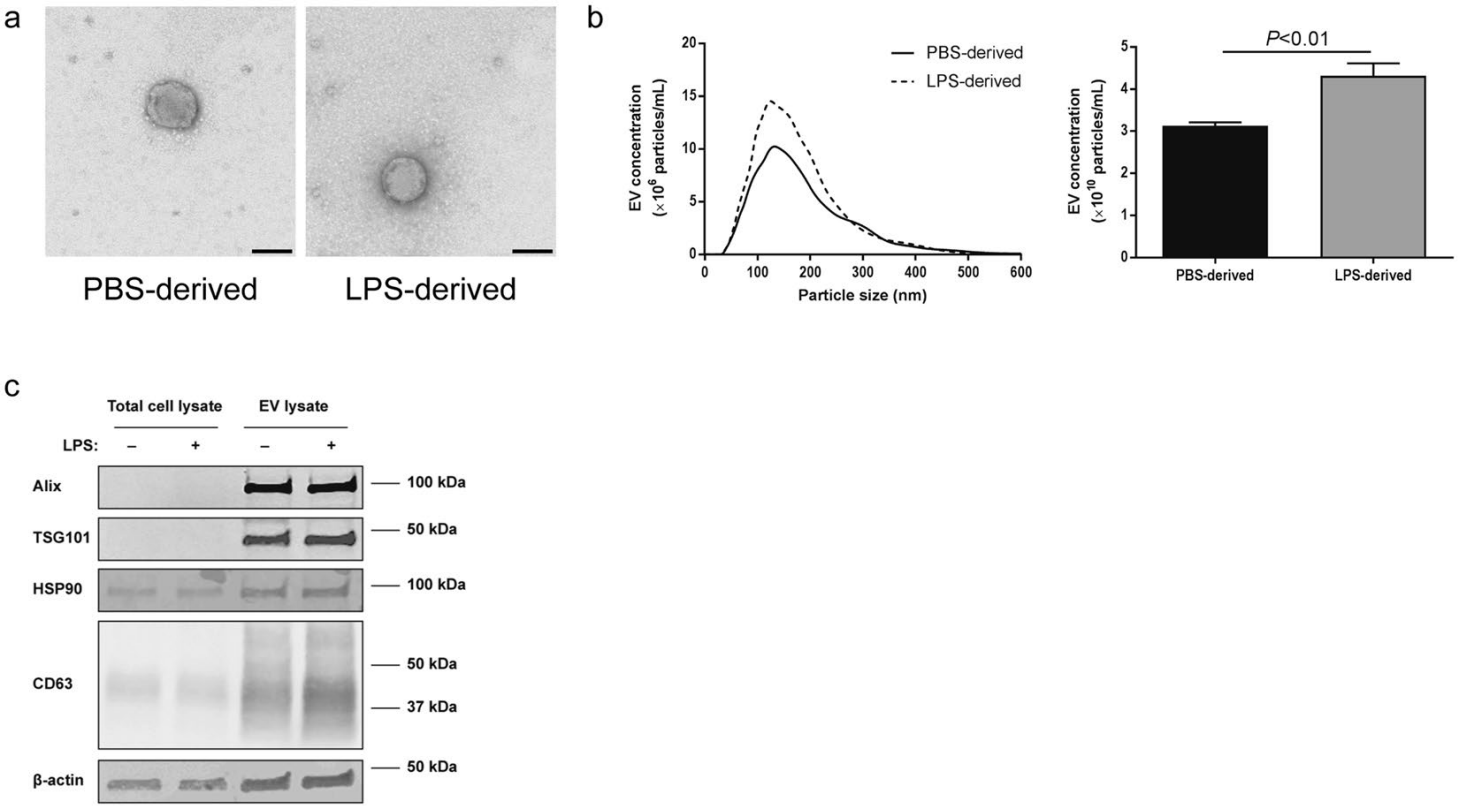
Isolation and identification of EVs secreted from H69 cells. (**a**) The morphology of PBS- and LPS-derived EVs analyzed by transmission electron microscope. Scale bar: 100 nm. (**b**) Nanoparticle tracking analysis for isolated H69-derived EVs. EV concentrations were calculated depending on the particle size. An example for PBS- (solid line) and LPS-derived (dashed line) EVs is shown (left). Total EV concentrations for PBS- and LPS-derived EVs (n = 6, right). (**c**) Immunoblotting for EV markers. Total H69 cell lysates and H69-derived EV lysates were analyzed by SDS-PAGE. The position of molecular weight markers for each protein is shown.

**Figure 2:**
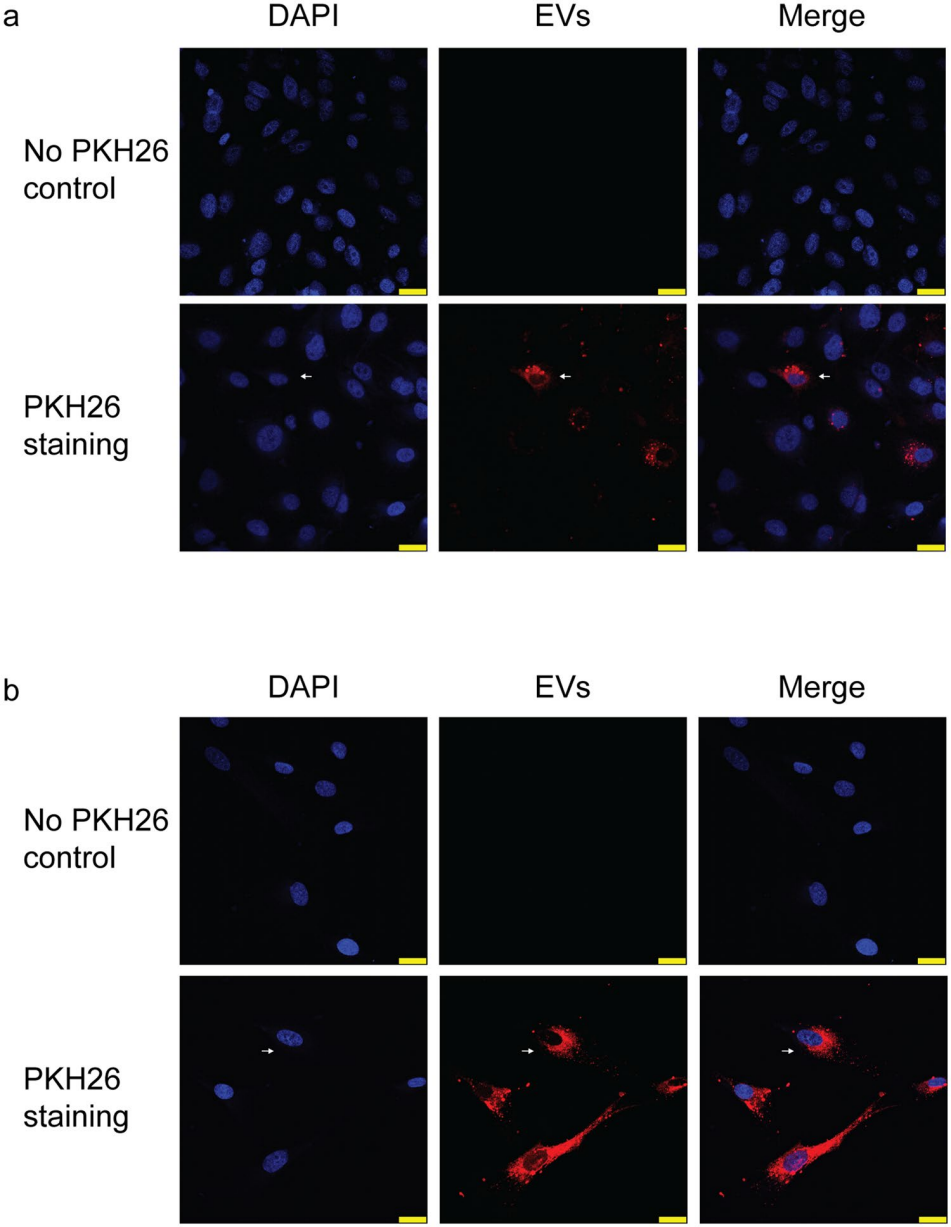
Internalization of H69-derived EVs into H69 cells or human primary hepatocytes. Internalization of H69-derived EVs into (**a**) other H69 cells and (**b**) human primary hepatocytes. Cell nuclei were stained by DAPI (blue) and H69-derived EVs were stained by PKH26 (red). EVs were merged with H69 cells or hepatocytes showing internalization (arrow). No PKH26 control is shown to eliminate possibility of unspecific binding of PKH26 or detection of background or noise. Scale bar: 25 µm.

Human H69 cells were stimulated with 1× PBS (vehicle) or LPS for 72 hours and culture media were harvested for EV isolation. Figure 1a shows the morphology of PBS- and LPS-derived EVs isolated from H69 cells. Nanoparticle tracking analysis (NTA) showed that the majority of particle size was 100–200 nm (Fig. 1b, left) and there was no difference in morphology and particle size between PBS- and LPS-derived H69 EVs. However, LPS-stimulated H69 cells secreted significantly (*P* < 0.01) higher numbers of total EVs compared to H69 cells treated with 1× PBS (Fig. 1b, right). Immunoblotting for EV markers showed clear bands for EV lysates proving successful EV isolation (Fig. 1c). No differences in expression levels for these EV markers were observed between PBS- and LPS-derived EVs. EV staining by PKH26 showed internalization of H69-derived EVs into other H69 cells demonstrating that H69 cells took up EVs and received their cargoes (Fig. 2a). EV internalization was also observed in human primary hepatocytes showing that hepatocytes can take up cholangiocyte EVs (Fig. 2b).

### LPS-derived EVs secreted from H69 cells induced inflammatory responses in other H69 cells, but not in hepatocytes

**Figure 3:**
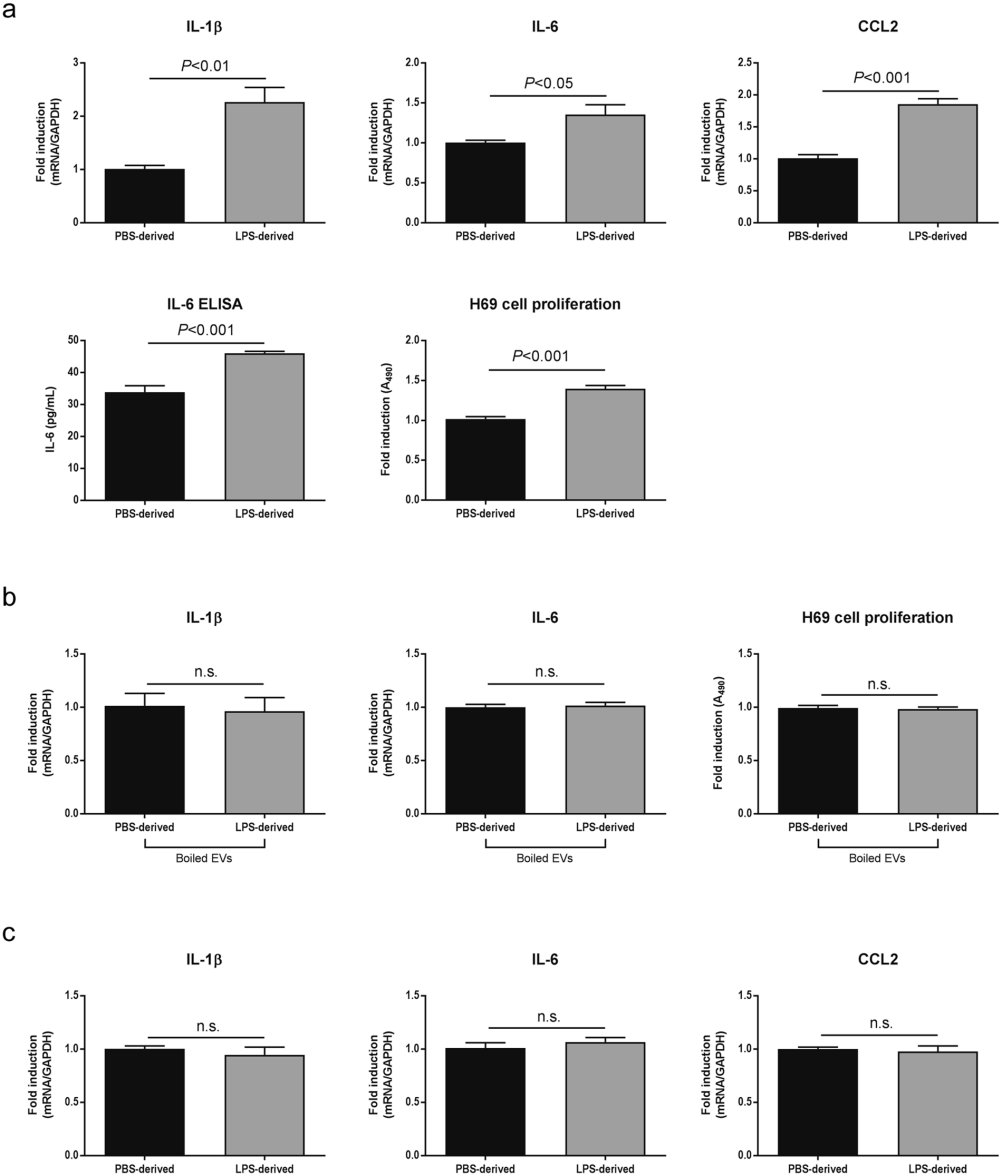
Inflammatory responses in H69 cells induced by LPS-derived EVs. (**a**) Analysis of inflammatory responses. H69 cells were incubated with PBS- or LPS- derived EVs for 48 hours. Total RNAs were harvested and two-step RT-PCR was performed (n = 5). For ELISA (n = 10) and cell proliferation assay (n = 10), H69 cells were cultured in 10 cm dishes or 96-well plates, respectively and incubated with EVs for 48 hours. Analyses were performed using commercial kits according to manufacturers’ instructions. (**b**) EV disruption abolished the effects of EVs. EVs were boiled at 95 °C for 15 min and incubated with H69 cells. After 48 hours, RT-PCR analysis for IL-1β and IL-6 (n = 3) and proliferation assay (n = 10) were performed. (**c**) H69-derived EVs did not induce inflammatory responses in primary hepatocytes. Human primary hepatocytes were incubated with PBS- or LPS-derived EVs isolated from H69 cells for 48 hours. RT-PCR was performed to analyze cytokine production (n = 10).

H69 cells were incubated with PBS- or LPS-derived EVs isolated from H69 cells for 48 hours. LPS-derived EVs induced mRNA expression of proinflammatory cytokines including IL-1β, IL-6, C-C motif chemokine ligand 2 (CCL2), which is also known as monocyte chemoattractant protein-1 (MCP-1). Significantly elevated (*P* < 0.001) IL-6 secretion was also detected by ELISA from culture media of H69 cells with LPS-derived EVs. H69 cell proliferation was significantly increased (*P* < 0.001) by LPS-derived EVs compared with PBS-derived EVs (Fig. 3a). EVs were disrupted by boiling at 95 °C and incubation of H69 cells with these disrupted EVs did not show elevated inflammatory responses in cytokine expression and cell proliferation (Fig. 3b) suggesting that inflammatory responses induced by LPS-derived EVs were caused by contents of EVs, not traces of LPS remaining after EV isolation. EVs were also disrupted by incubation at 4 °C for one week^24^, and these disrupted EVs also did not induce H69 cell proliferation (not shown). Interestingly, LPS-derived EVs isolated from H69 cells did not induce proinflammatory cytokine production in human primary hepatocytes (Fig. 3c), indicating that hepatocytes may not participate in inflammatory cholangiocyte communication even if they internalize with cholangiocyte-derived EVs.

### Inflammatory responses induced by LPS-derived EVs depend on the heterogeneity of cholangiocytes

**Figure 4:**
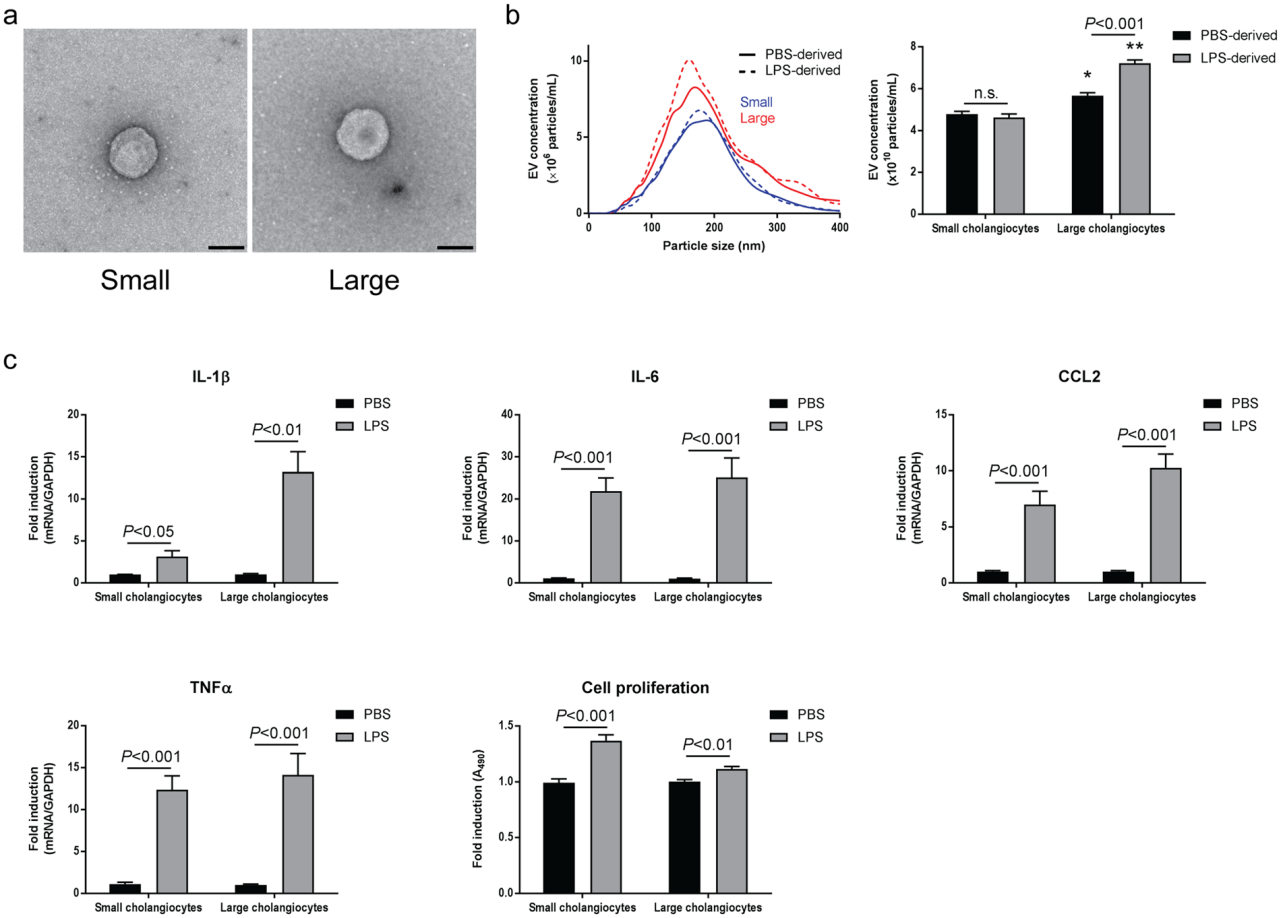
Secretion of EVs and responses against LPS in murine small and large cholangiocytes. (**a**) The morphological analysis by transmission electron microscope for PBS-derived EVs isolated from small (left) and large (right) cholangiocytes. Scale bar: 100 nm. (**b**) Nanoparticle tracking analysis for EVs secreted from small and large cholangiocytes. An example for PBS- (solid lines) and LPS-derived (dashed lines) EVs isolated from small (blue lines) and large (red lines) cholangiocytes is shown (left). Total EV concentrations are shown (right). *P < 0.05, **P < 0.001 vs. small cholangiocytes with same stimulation (n = 5). (**c**) Inflammatory responses against LPS in small and large cholangiocytes. For RT-PCR of proinflammatory cytokines, small and large cholangiocytes were incubated with 1× PBS or 200 ng/mL LPS for 3 hours. Total RNAs were harvested and two-step RT-PCR was performed (n = 6). For cell proliferation assay, small and large cholangiocytes were incubated with 1× PBS or 200 ng/mL LPS for 48 hours (n = 10).

There was no difference in the morphology of isolated PBS-derived EVs between small and large cholangiocytes (Fig. 4a). NTA showed that the majority of particle size was 100–200 nm (Fig. 4b, left) and there was no difference in particle size between small and large EVs. However, large cholangiocytes secreted significantly (*P* < 0.05) more total EVs than small cholangiocytes, and LPS stimulation increased EV secretion from large but not small cholangiocytes (Fig. 4b, right). Firstly, small and large cholangiocytes were stimulated with 1× PBS or LPS to evaluate direct responses of these cells against LPS. LPS enhanced proinflammatory cytokine expression as well as cell proliferation in both small and large cholangiocytes (Fig. 4c).

**Figure 5:**
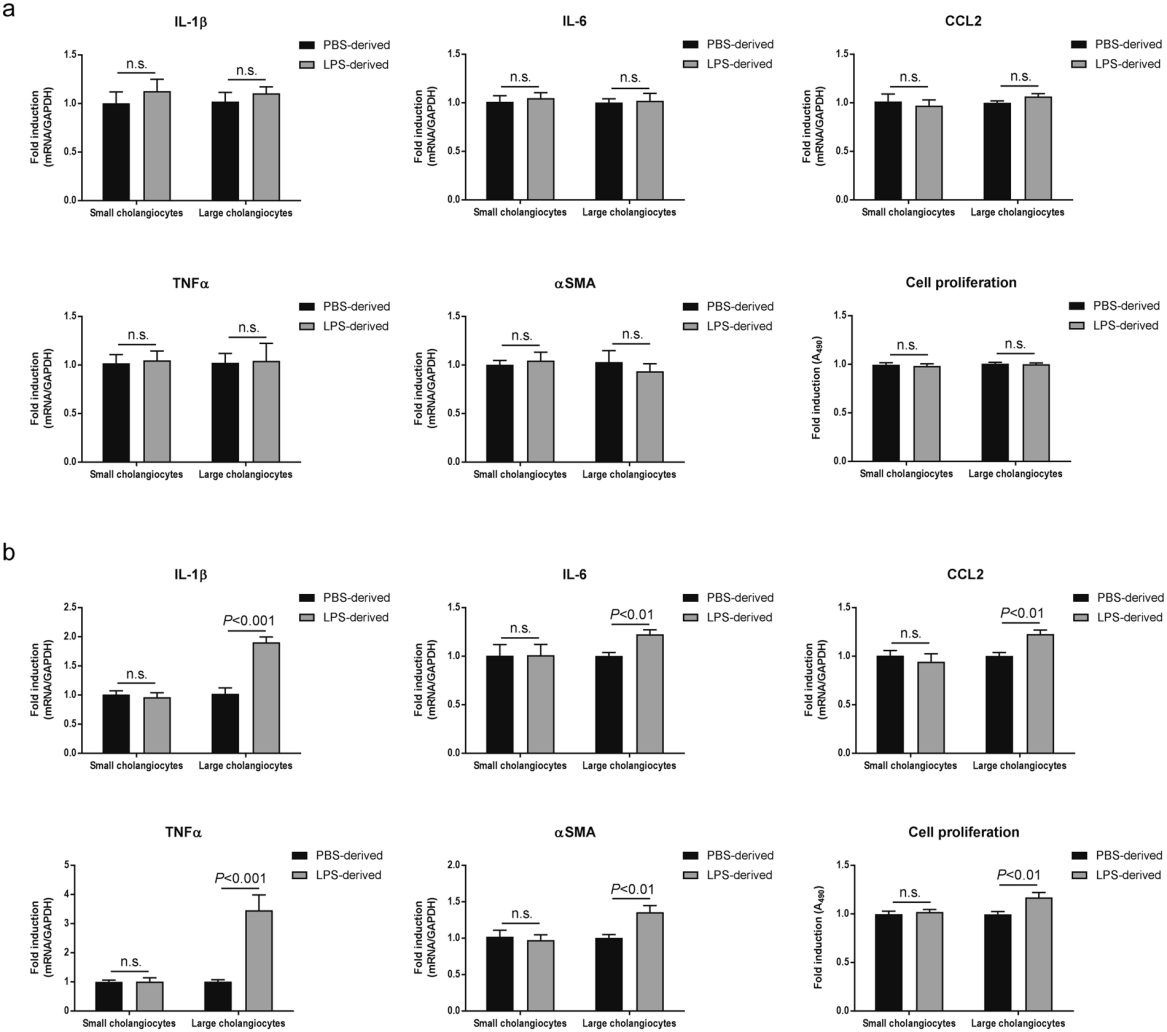
Inflammatory cell-cell communication between large cholangiocytes. (**a**) Small and large cholangiocytes were incubated with PBS- or LPS-derived EVs isolated from small cholangiocytes for 48 hours. RT-PCR for proinflammatory cytokine expression (n = 6) and cell proliferation assay (n = 10) were performed. (**b**) Small and large cholangiocytes were incubated with EVs isolated from large cholangiocytes for 48 hours. RT-PCR (n = 6) and cell proliferation assay (n = 10) were performed.

Secondly, small or large cholangiocytes were incubated with EVs isolated from small cholangiocytes. RT-PCR did not detect elevated expression for all cytokines including IL-1β, IL-6, CCL2 and tumor necrosis factor α (TNFα) as well as fibrogenesis marker α-smooth muscle actin (αSMA), induced by LPS-derived small EVs. Cell proliferation assay also showed no effects of small-derived EVs in both small and large cholangiocytes (Fig. 5a).

Thirdly, LPS-derived EVs isolated from large cholangiocytes induced proinflammatory cytokine production, αSMA expression, and cell proliferation in large cholangiocytes, but not in small cholangiocytes (Fig. 5b). The inflammatory cell-cell communication induced by LPS stimulation was identified only between large cholangiocytes in experimental conditions of this study.

### The SCT/SR axis is required to LPS-induced cell-cell communication

**Figure 6:**
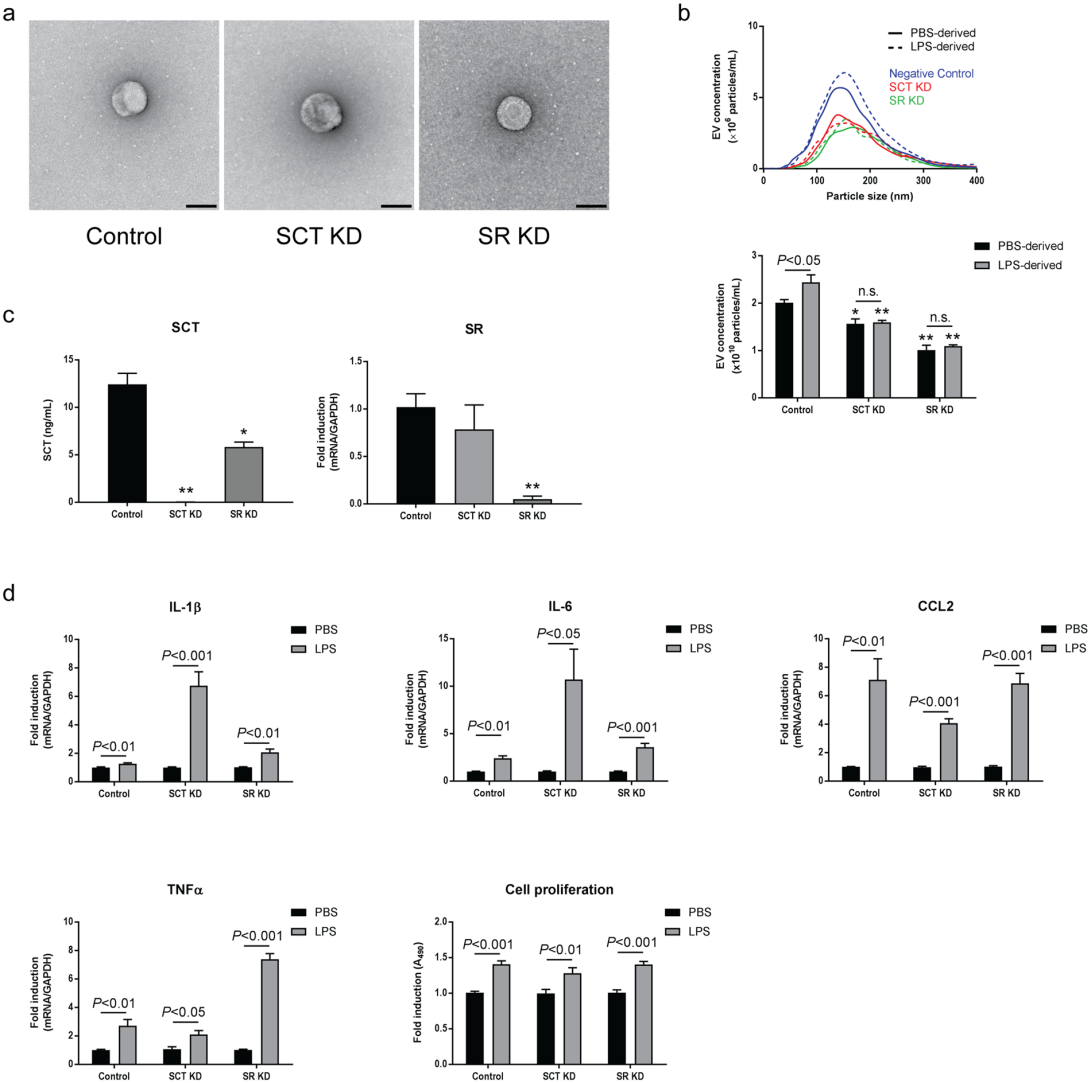
Secretion of EVs and responses against LPS in large cholangiocytes with SCT or SR knockdown. (**a**) The morphological analysis by transmission electron microscope for EVs isolated from control large cholangiocytes (left), with SCT knockdown (middle), and with SR knockdown (right). Scale bar: 100 nm. (**b**) Nanoparticle tracking analysis for isolated EVs. An example for PBS- (solid lines) and LPS-derived (dashed lines) EVs isolated from control (blue lines), SCT knockdown (red lines), and SR knockdown (green lines) cholangiocytes is shown (top). Total EV concentrations are shown (bottom). *P < 0.05, **P < 0.001 vs. control cholangiocytes with same stimulation (n = 5). (**c**) Knockdown efficiencies for SCT and SR. Control, SCT or SR knockdown cholangiocytes were incubated with 50 ng/mL LPS for 72 hours in 6-well plates. Culture media were harvested for ELISA for SCT, and total RNAs were harvested from cells for RT-PCR for SR. *P < 0.05, **P < 0.001 vs. control cholangiocytes (n = 3). (**d**) For RT-PCR for proinflammatory cytokine expression, cholangiocytes were incubated with 1× PBS or 200 ng/mL LPS for 3 hours. Total RNAs were harvested and two-step RT-PCR was performed (n = 6). For cell proliferation assay, cholangiocytes were incubated with 1× PBS or 200 ng/mL LPS for 48 hours (n = 10).

No morphological differences were observed between PBS-derived EVs isolated from mock-transfected control large cholangiocytes, large cholangiocytes with SCT knockdown, and large cholangiocytes with SR knockdown (Fig. 6a). No differences in particles size were also observed by NTA (Fig. 6b, top). Control cholangiocytes secreted significantly higher numbers of EVs during LPS stimulation as observed in previous experiments using large cholangiocytes. However, large cholangiocytes with SCT or SR knockdown secreted significantly less EVs than control large cholangiocytes and LPS stimulation did not increase EV secretion from these cells (Fig. 6b, bottom).

ELISA for SCT and RT-PCR for SR showed efficient knockdown of SCT and SR in corresponding knockdown cells (Fig. 6c). Control, SCT knockdown, or SR knockdown large cholangiocytes were stimulated with 1× PBS or LPS. As well as small and large cholangiocytes, these cells showed strong responses against LPS in cell proliferation and cytokine production regardless of gene knockdown (Fig. 6d).

**Figure 7:**
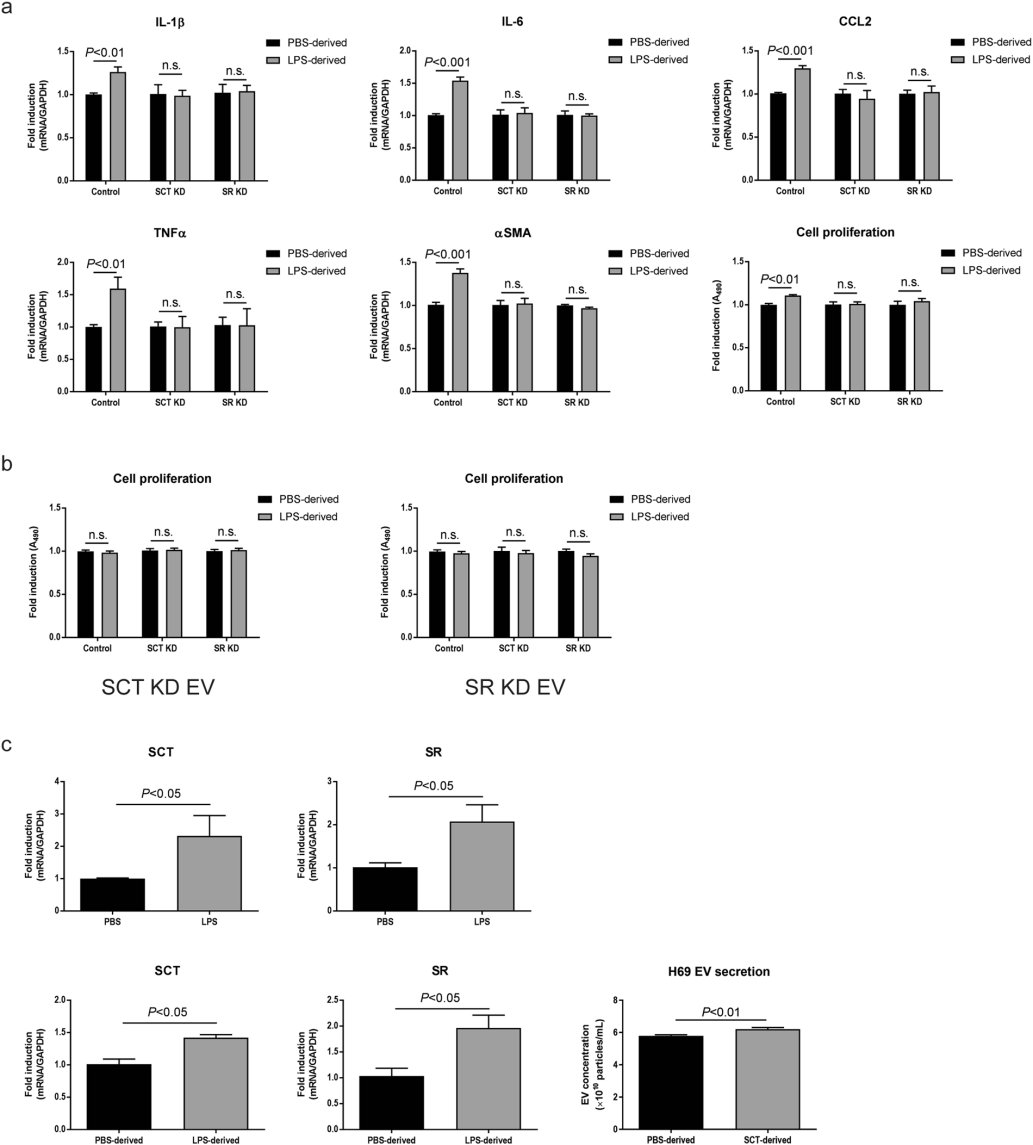
The SCT/SR axis is required for cholangiocyte communication via LPS-derived EVs. (**a**) Effects of control EVs. Cholangiocytes were incubated with PBS- or LPS-derived EVs isolated from control large cholangiocytes for 48 hours. Expression of proinflammatory cytokines and αSMA was analysed by RT-PCR (n = 6). Cell proliferation assay is also shown (n = 10). (**b**) Effects of SCT or SR knockdown EVs. Cholangiocytes were incubated with EVs isolated from SCT knockdown (left) or SR knockdown (right) cells, and cell proliferation assay was performed (n = 10). (**c**) Association between LPS stimulation and the SCT/SR axis. H69 cells were stimulated with 50 ng/mL LPS or LPS-derived H69 EVs for 72 hours and RT-PCR was performed. Both LPS (top) and LPS-derived EVs (bottom) elevated expression levels of SCT and SR in H69 cells (n = 5). H69 cells were stimulated with 100 nM SCT for 72 hours and EVs were then harvested. Nanoparticle tracking analysis was performed for total particle concentrations of PBS- or SCT-derived H69 EVs (n = 5, bottom right).

Cells were incubated with PBS- or LPS-derived EVs isolated from control cholangiocytes. LPS-derived control EVs elevated proinflammatory cytokine production, αSMA expression, and cell proliferation in control cholangiocytes, but not in SCT or SR knockdown cells (Fig. 7a). EVs isolated from SCT or SR knockdown cholangiocytes failed to elevate cell proliferation of all cholangiocytes including control, suggesting that LPS-derived cell-cell EV communication can occur only between control EVs and control cholangiocytes (Fig. 7b).

To explore the association between LPS stimulation and the SCT/SR axis, H69 cells were stimulated by LPS for 48 hours. LPS stimulation induced both SCT and SR expression in H69 cells. In addition, LPS-derived H69 EVs also induced SCT and SR expression in other H69 cells after 48-hour incubation. As well as LPS stimulation, SCT stimulation elevated EV secretion in H69 cells (Fig. 7c), suggesting that LPS stimulation is associated with SCT/SR expression and SCT stimulation is associated with EV secretion. Taken together, these results suggest that the SCT/SR axis is required for effective EV secretion and secretion of functional EVs needed for LPS-induced inflammatory cell-cell communication between large cholangiocytes.

## Discussion

EVs have attracted great interests in recent research fields of liver diseases^20, 21^. Accumulating studies suggest that the cell-cell communication via EVs play a key role in pathophysiology of liver diseases. Lipids increase EV secretion from hepatocytes and these lipid-derived EVs activate liver macrophages leading to elevated expression of proinflammatory cytokines including IL-6^25^. Alcohol treatments also elevate EV secretion from hepatocytes as well as proinflammatory cytokine production from macrophages induced by alcohol-derived EVs^26^. These studies suggest that the cell-cell communication between hepatocytes and macrophages may be responsible for liver damage in alcoholic and non-alcoholic liver diseases. In cholestatic liver diseases, however, the cell-cell communication as well as EV secretion and its functions are largely unknown. The present study identified for the first time the cell-cell communication between cholangiocytes via EV secretion during LPS stimulation, a model of bacterial infection.

LPS stimulation increased EV secretion from H69 cells (Fig. 1b) and mouse large cholangiocytes as well as mock-transfected large cholangiocytes (Figs 4b and 6b) in this study. Previous studies showed that activated hepatocytes induced by alcohol or lipids secreted more EVs than unstimulated hepatocytes^25, 26^. As activated hepatocytes, activated cholangiocytes induced by LPS also secrete more EVs than unstimulated cholangiocytes to communicate with other cholangiocytes and to induce inflammatory responses as a counter reaction against bacterial infection. A previous study has shown that LPS stimulation induces H69 cell proliferation via IL-6 production^5^, and hence this might also contribute to increased EV secretion from cholangiocytes during LPS stimulation observed in this study.

Small cholangiocytes as well as large cholangiocytes with SCT or SR knockdown secreted less EVs than large or control cholangiocytes, respectively (Figs 4b and 6b). Small cholangiocytes may be physically unable to secrete as many EVs as large cholangiocytes because they have a high nucleus/cytoplasm ratio whereas large cholangiocytes have a small nucleus and conspicuous cytoplasm^13, 27, 28^. In addition, a previous study has shown that SCT induces exocytosis from cholangiocytes by a cAMP-dependent mechanism^29^. Other studies have shown that cholangiocyte proliferation and functions are regulated by a number of stimulatory hormonal factors including SCT in an autocrine/paracrine fashion^2, 11, 30, 31^. Our study has shown that not only SCT knockdown cholangiocytes, but also SR knockdown cells secrete significantly reduced SCT compared to control cholangiocytes (Fig. 6c), suggesting the close relationship between SCT and SR in cholangiocyte functions. This study has also shown that SCT increases EV secretion from H69 cells (Fig. 7c). These findings suggest that heterogeneity of cholangiocytes and the SCT/SR axis are associated with EV secretion, and small cholangiocytes that lack SR or large cholangiocytes with SCT or SR knockdown cannot perform effective EV secretion. LPS-derived EVs isolated from these cells did not induce inflammatory responses in large cholangiocytes or control cholangiocytes (Figs 5 and 7). LPS stimulation is associated not only with EV secretion but also with SCT and SR expression in cholangiocytes (Fig. 7c). These findings suggest that the SCT/SR axis is critical for LPS-induced inflammatory EV communication between cholangiocytes.

LPS binds to Toll-like receptor 4 (TLR4) and activates the nuclear-factor κB (NF-κB) pathway leading to immune responses in cholangiocytes^32^. Small cholangiocytes and large cholangiocytes with SCT or SR knockdown showed responses against LPS in cell proliferation and cytokine production (Figs 4c and 6d). Association between the SCT/SR axis and the TLR4-NF-κB pathway is still unclear, and further studies are required to elucidate detailed mechanisms of immune responses induced by LPS-derived EVs in control large cholangiocytes.

It is known that both hepatocytes and cholangiocytes respond to LPS and secrete proinflammatory cytokines such as IL-6^5, 6, 33, 34^. LPS-derived EVs isolated from H69 cells induced inflammatory responses in other H69 cells but not in primary hepatocytes in this study (Fig. 3). Cholangiocyte-derived EVs may induce responses in other liver cells, such as macrophages or neutrophils, which are associated with liver inflammation. This study is not conclusive that the cell-cell communication identified in this study is specific between cholangiocytes. Further studies are also required to elucidate the mechanism that mediators carried in LPS-derived EVs induce inflammatory responses in H69 cells but not in hepatocytes.

Depletion of EVs abolished the effects of LPS-derived EVs in H69 cells (Fig. 3c), and LPS-derived EVs from small cholangiocytes or large cholangiocytes with SCT or SR knockdown did not induce any response in large or control cholangiocytes, respectively (Figs 5a and 7b). These observations suggest that inflammatory responses were caused by cargoes of LPS-derived EVs, not traces of LPS remaining after EV isolation.

This study did not identify cell-cell communication between small and large cholangiocytes. It is probably because small cholangiocytes do not have the SCT/SR axis, which is required for effective EV secretion and inflammatory responses. Previous studies, however, suggest that small cholangiocytes are hepatic progenitor cells and can differentiate into large cholangiocytes when large bile ducts are damaged^18, 19^. It is highly possible that small cholangiocytes receive signals from damaged large cholangiocytes via EVs and start differentiation. Further studies are required for EV communication between small and large cholangiocytes.

Even though this study presents novel molecular mechanisms for human cholestatic liver injuries, it also has several limitations. First, this is an *in vitro* study and cells were cultured in flasks, and hence apical or basolateral domains were not distinguished. Cholangiocytes line to form bile ducts and these two regions express different proteins and have different functional roles. The basolateral region expresses receptors such as SR to detect autocrine and paracrine mediators, and the apical region has a primary cilium to detect biliary exosomes and other mediators^23, 28, 35^. It is possible that EVs secreted from apical domains have different functions from those from basolateral domains. This study used the mixture of EVs secreted from any regions of cholangiocytes. Second, this study focused on the functional effects of EVs, not EV cargoes. Further studies are required to conclude which mediators, proteins, mRNAs, microRNAs or proinflammatory cytokines, were transferred via EVs leading to inflammatory responses.

In conclusion, cholangiocytes communicate each other via EV secretion during LPS-induced inflammation, and the SCT/SR axis may be important for EV secretion and functional signal transduction during this event.

## Methods

### Cell culture

H69 cells, immortalized human normal cholangiocytes (a gift from Dr. G. J. Gores, Mayo Clinic, MN), were maintained as described^36, 37^. Our immortalized small and large cholangiocytes were also maintained as described^7, 11, 14, 38^. Stable transfected knockdown of SCT or SR in murine large cholangiocyte lines using short hairpin RNA (shRNA) plasmids were established in our laboratory and maintained as described^7, 11^. Human primary hepatocytes and culture media for these cells were purchased from ScienCell Research laboratories (Carlsbad, CA) and maintained according to the manufacturer’s instruction. Cells (3 × 10^5^) were cultured in T175 flasks containing 30 mL of culture media with 1× phosphate buffered saline (PBS), LPS (50 ng/mL, Sigma-Aldrich, St Louis, MO), or SCT (100 nM, Bachem, Bubendorf, Switzerland) for 72 hours. Supplement fetal bovine serum (FBS) was depleted of EVs by ultracentrifugation at 120,000 × g for 18 hours (Type 50.2 Ti rotor, Beckman Coulter, Brea, CA)^39^. All cells were cultured at 37 °C and 5% CO_2_.

### Isolation of EVs

EVs were isolated from culture media as described with minor changes^40, 41^. Briefly, 30 mL of culture media were harvested and centrifuged at 300 × g for 10 minutes to remove cells followed by 30 minutes at 3,000 × g to remove cells debris, and supernatants were filtered through 0.22 µm syringe filters. EVs containing exosomes and small microvesicles were pelleted by ultracentrifugation at 120,000 × g for 3 hours. EV pellets were resuspended and washed in 30 mL of 1× PBS. EVs from H69 cells were then pelleted again by ultracentrifugation at120,000 × g for 2 hours, and then resuspended in 500 µL of 1× PBS. EV pellets from mouse cholangiocytes were merged with 2-3 samples during 1× PBS washes due to low amounts of EVs obtained, which means that EVs were isolated from 30 mL culture media for H69 cells and from 60-90 mL for murine cholangiocytes. All procedures were performed on ice or at 4 °C.

### Characterization of isolated EVs

EV concentrations in 1× PBS were determined by nanoparticle tracking analysis using NanoSight LM10HS (Malvern Instruments, Malvern, UK). The morphology of EVs were examined by negative staining and electron microscopy. EVs in 1× PBS were fixed in 1% glutaraldehyde and allowed to absorb onto formvar/carbon-coated copper grids for 10 min. Grids were washed with water and stained with 1% aqueous uranyl acetate (Ted Pella Inc., Redding, CA) for 1 min. Excess liquid was gently wicked off and grids were allowed to air dry. Samples were viewed on a JEOL 1200EX transmission electron microscope (JEOL USA, Peabody, MA) equipped with an AMT 8 megapixel camera (Advanced Microscopy Techniques, Woburn, MA).

### Immunoblotting for EV markers

Cells were pelleted by centrifugation after harvesting culture media for EV isolation, and cell pellets or EV pellets were resuspended in RIPA buffer (ThermoFisher Scientific, Waltham, MA) supplemented with protease inhibitors. Cells and EVs were disrupted using the ultrasonic sonicator and solubilized for 20 minutes on ice. Protein concentrations were determined using Pierce BCA Protein Assay Kit (ThermoFisher Scientific). Total proteins (2 µg) were separated by SDS-PAGE on 4-20% Mini-PROTEAN TGX gel (Bio-Rad, Hercules, CA) and transferred to a nitrocellulose membrane (LI-COR Bioscience, Lincoln, NE). After blocking the membrane with Odyssey Blocking Buffer (LI-COR Bioscience), proteins of interest were detected by primary and secondary antibodies. We used the following antibodies: (i) anti-β actin (Sigma-Aldrich); (ii) anti-Alix and anti-HSP90 (Cell Signaling Technology, Danvers, MA); (iii) anti-TSG101 (abcam, Cambridge, MA); and (iv) anti-CD63 (ThermoFisher Scientific). Secondary antibodies were purchased from LI-COR Bioscience. Protein bands were detected using LI-COR Odyssey Infrared Imaging System (LI-COR Bioscience).

### Immunofluorescent detection for EV internalization

H69-derived EVs were pelleted as described above. EVs were stained using PKH26 Red Fluorescent Cell Linker Mini Kit for General Cell Membrane Labeling (Sigma-Aldrich) according to the manufacturer’s instruction. Five thousand H69 cells or human primary hepatocytes were cultured on a glass cover slip in a 6-well plate. Cells were incubated with stained or unstained H69-derived EVs (1 × 10^8^ particles/mL) for 24 hours. Glass cover slips with cells were washed with 1× PBS and fixed in 4% formaldehyde for 20 minutes. EV internalization was observed using Leica TCS SP5 X confocal microscope (Leica Microsystems, Buffalo Grove, IL).

### Analysis of inflammatory responses induced by EVs

Human primary hepatocytes or H69 cells, murine small, large, or gene-knocked down cholangiocytes (1 × 10^4^) were cultured in 6-well plates. Cells were incubated with PBS- or LPS-derived EVs (5 × 10^8^ particles/mL) for 48 hours. Total RNAs were harvested using RNeasy Mini Kit (QIAGEN, Valencia, CA). Two-step RT-PCR was performed using RT2 qPCR Primer Assay (QIAGEN) and ViiA 7 Real-Time PCR System (ThermoFisher Scientific). PCR primers used in this study are listed in Table 1. For IL-6 ELISA, H69 cells (1 × 10^5^) were cultured in 10 cm dishes and incubated with EVs (2 × 10^8^ particles/mL) for 48 hours. IL-6 in culture media was detected using Human IL-6 ELISA Kit II (BD Biosciences, San Jose, CA). SCT in culture media was detected using Mouse EIA Kit (Phoenix Pharmaceuticals, Burlingame, CA). For cell proliferation assay, 200-1,000 cells were cultured in 96-well plate and incubated with EVs (1 × 10^9^ particles/mL) for 48 hours. Cell proliferation was analysed using CellTiter 96 AQ_ueous_ One Solution Cell Proliferation Assay (Promega Corporation, Madison, WI) according to the manufacturer’s instruction. To disrupt EVs, EVs were boiled at 95 °C for 15 min or incubated at 4 °C for 1 week^24^ Murine cholangiocytes were also incubated with 1× PBS or 200 ng/mL LPS for 3 hours to evaluate the direct response against LPS.

**Table 1:** Primers used for RT-PCR in this study (QIAGEN RT2 Primer Assay).

Gene	Accession Number	Catalogue Number
**Primers for human H69 cells**		
GAPDH	NM_001256799	PPH00150F
IL-1β	NM_000576	PPH00171C
IL-6	NM_000600	PPH00560C
CCL2	NM_002982	PPH00192F
SCT	NM_021920	PPH60555B
SR	NM_002980	PPH18173F
**Primers for murine cells**		
GAPDH	NM_008084	PPM02946E
IL-1β	NM_008361	PPM03109F
IL-6	NM_031168	PPM03015A
CCL2	NM_011333	PPM03151G
TNFα	NM_013693	PPM03113G
αSMA	NM_007392	PPM04483A
SR	NM_001012322	PPM32676F

### Statistical analysis

Data were expressed as mean ± SEM. Statistical significance of differences between control and experimental groups was analysed by unpaired Student’s *t*-test using GraphPad Prism 7 (GraphPad Software, La Jolla, CA). Values of *P* < 0.05 were considered as statistically significant.

### Data Availability

The datasets generated during and/or analysed during the current study are available from the corresponding author on reasonable request.

## Electronic supplementary material

Supplementary info
