# Author Correction: The role of the secretin/secretin receptor axis in inflammatory cholangiocyte communication via extracellular vesicles

**DOI:** 10.1038/s41598-018-29609-x

**Published:** 2018-07-20

**Authors:** Keisaku Sato, Fanyin Meng, Julie Venter, Thao Giang, Shannon Glaser, Gianfranco Alpini

**Affiliations:** 1 Research, Central Texas Veterans Health Care System, Temple, TX 76504 USA; 2 Department of Medicine, Texas A&M College of Medicine, Temple, TX 76504 USA; 3 Baylor Scott & White Digestive Disease Research Center, Baylor Scott & White Healthcare, Temple, TX 76504 USA; 4 Academic Research Integration, Baylor Scott & White Healthcare, Temple, TX 76504 USA

Correction to: *Scientific Reports* 10.1038/s41598-017-10694-3, published online 11 September 2017

This Article contains typographical errors in the Acknowledgements section.

“This work was supported by the Dr. Nicholas C. Hightower Centennial Chair of Gastroenterology from Baylor Scott & White, a VA Research Career Scientist Award and a VA Merit award to Dr. Alpini (5I01BX000574), a VA Merit Award (5I01BX002192) to Dr. Glaser, a VA Merit Award (1I01BX001724) to Dr. Meng, and the NIH grants DK058411, DK076898, DK095291 and DK062975 to Drs. Alpini, Meng and Glaser.”

should read:

“This work was supported by the Dr. Nicholas C. Hightower Centennial Chair of Gastroenterology from Baylor Scott & White, a VA Research Career Scientist Award and a VA Merit award to Dr. Alpini (5I01BX000574), a VA Merit Award (5I01BX002192) to Dr. Glaser, a VA Merit Award (1I01BX001724) to Dr. Meng, and the NIH grants DK058411, DK076898, DK107310 and DK062975 to Drs. Alpini, Meng and Glaser.”

