# Supplementary material for: The role of the secretin/secretin receptor axis in inflammatory cholangiocyte communication via extracellular vesicles
Source: Sci Rep. 2017 Sep 11;7:11183. doi: 10.1038/s41598-017-10694-3 (PMC5593902; doi:10.1038/s41598-017-10694-3)
Supplement: Supplementary file 1 — Supplementary info [file 41598_2017_10694_MOESM1_ESM.pdf]

The role of the secretin/secretin receptor axis in inflammatory cholangiocyte communication via extracellular vesicles

Keisaku Sato[1,2,3], Fanyin Meng[1,2,3,4,*], Julie Venter[1,2,3], Thao Giang[1,2,3], Shannon Glaser[1,2,3*], Gianfranco Alpini[1,2,3*]

From [1]Research, Central Texas Veterans Health Care System, [2]Department of Medicine, Texas A&M College of Medicine, [3]Baylor Scott & White Digestive Disease Research Center, Baylor Scott & White, and [4]Academic Research Integration, Baylor Scott & White Healthcare, Temple, TX 76504.

[*]Corresponding authors: Fanyin Meng, FMeng@medicine.tamhsc.edu; Shannon Glaser, SGlaser@medicine.tamhsc.edu; Gianfranco Alpini, GAlpini@medicine.tamhsc.edu;

[*]Drs. Meng, Glaser and Alpini share the senior authorship.

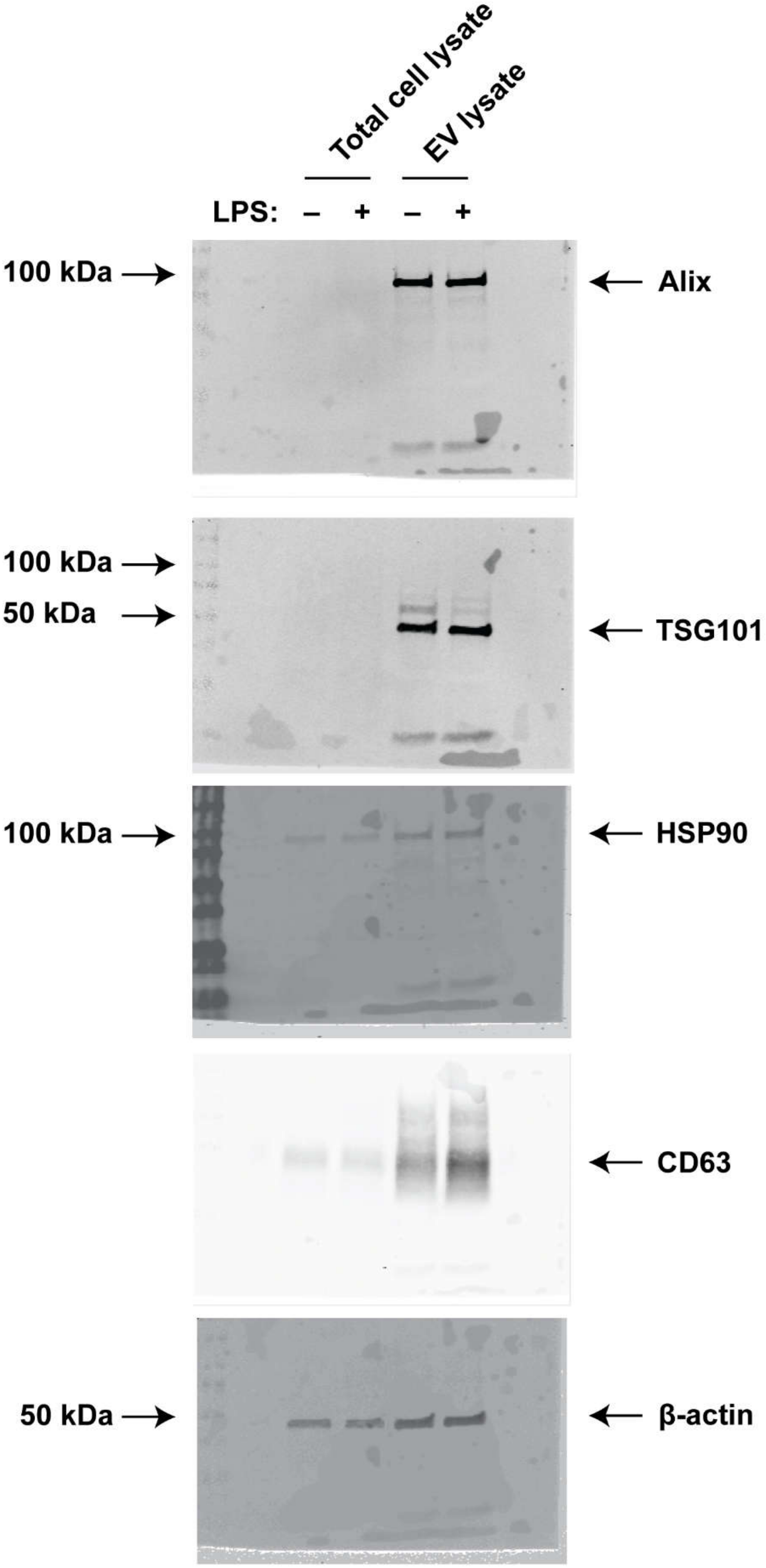
Total cell lysate
EV lysate
LPS: – + – +
100 kDa
Alix
100 kDa
50 kDa
TSG101
100 kDa
HSP90
CD63
50 kDa
β-actin
